# Characterization of *Fatty Acid Exporters* involved in fatty acid transport for oil accumulation in the green alga *Chlamydomonas reinhardtii*

**DOI:** 10.1186/s13068-018-1332-4

**Published:** 2019-01-12

**Authors:** Nannan Li, Yan Zhang, Hongjun Meng, Shengting Li, Shufeng Wang, Zhongchun Xiao, Peng Chang, Xiaohui Zhang, Qing Li, Liang Guo, Yasuo Igarashi, Feng Luo

**Affiliations:** 1 Research Center of Bioremediation and Bioenergy, College of Resources and Environment, Southwest University, Beibei, Chongqing, 400715 People’s Republic of China; 2 Academy of Agricultural Science, Southwest University, Beibei, Chongqing, 400715 People’s Republic of China; 3 National Key Laboratory of Crop Genetic Improvement, Huazhong Agricultural University, Wuhan, 430070 China

**Keywords:** Fatty acid exporter, Gene overexpression, Transcriptomic analysis, Triacylglycerol, *Chlamydomonas reinhardtii*

## Abstract

**Background:**

In the past few decades, microalgae biofuel has become one of the most interesting sources of renewable energy. However, the higher cost of microalgae biofuel compared to that of petroleum prevented microalgae biofuel production. Therefore, the research on increasing lipid productivity from microalgae becomes more important. The lipid production source, triacylglycerol biosynthesis in microalgae requires short chain fatty acids as substrates, which are synthesized in chloroplasts. However, the transport mechanism of fatty acids from microalgae chloroplasts to cytosol remains unknown.

**Results:**

cDNAs from two homologs of the Arabidopsis fatty acid exporter 1 (FAX1) were cloned from *Chlamydomonas reinhardtii* and were named *crfax1* and *crfax2.* Both CrFAXs were involved in fatty acid transport, and their substrates were mainly C16 and C18 fatty acids. Overexpression of both CrFAXs increased the accumulation of the total lipid content in algae cells, and the fatty acid compositions were changed under normal TAP or nitrogen deprivation conditions. Overexpression of both CrFAXs also increased the chlorophyll content. The MGDG content was decreased but the TAG, DAG, DGDG and other lipid contents were increased in CrFAXs overexpression strains.

**Conclusion:**

These results reveal that CrFAX1 and CrFAX2 were involved in mediating fatty acid export for lipids biosynthesis in *C. reinhardtii*. In addition, overexpression of both CrFAXs obviously increased the intracellular lipid content, especially the triacylglycerol content in microalgae, which provides a potential technology for the production of more biofuels using microalgae.

**Electronic supplementary material:**

The online version of this article (10.1186/s13068-018-1332-4) contains supplementary material, which is available to authorized users.

## Background

In the last few decades, microalgae have been regarded as a powerful raw material for the production of biofuel, the most promising renewable energy source. Microalgae can photoautotrophically grow and produce bulk chemicals, such as triacylglycerol (TAG) and starch, which can be transformed into biodiesel and bioethanol, respectively [1]. However, the lipid content of microalgae needs to be increased for commercial-scale production of biodiesel from microalgae [2, 3]. Therefore, increasing the lipid content in microalgae through genetic engineering techniques is needed to improve the potential commercial production of biodiesel from microalgae.

In recent years, research on lipid metabolism in plants has characterized many key genes in the triacylglycerol synthesis pathways [4, 5]. In plants and algae, fatty acids (FAs) are uniquely synthesized in plastids, which is different from other prokaryotic and eukaryotic cells. Most of the synthesized FAs are then transported to the endoplasmic reticulum (ER) for modification and lipid assembly. Subsequently, lipids, such as TAGs in seeds, are used for lipid mobilization, which is important for seed germination [6]. The membrane-localized transporters and proteins required for cellular lipid transport play key roles in lipid metabolism in plant and algae cells. In the past few decades, many key transporters have been identified in plants. The chloroplast inner envelope localized AtFAX1 (Fatty Acid EXporter 1) mediates fatty acid export from plastids [5, 7]. In addition, the outer envelope of plastid-localized long-chain acyl-CoA synthetase 9 (AtLACS9) may drive acylation for plastid FA export [8, 9]. One of the ATP-binding cassette (ABC) transporters of subfamily A, AtABCA9, was shown to import FA/acyl-CoA into the ER [10]. The FA β-oxidation process occurs in plant peroxisomes, and FAs import into the peroxisomes is mediated by full-size ABCD1 transporters [11–13].

However, until recently, fatty acid and lipid biosynthesis in microalgae has been rarely studied [5, 14–18]. The general framework of the metabolic networks involved in TAG synthesis in microalgae can be deduced based on orthologs of the proteins of plant lipid metabolism. Members of the major protein families, such as ABCA and ABCD transporters, ACBP, and LACS, involved in FA/lipid transport in plants are also encoded in the genome of *Chlamydomonas reinhardtii* (*C. reinhardtii*), which is the best studied model green alga for transgenic research [10, 12, 13, 19–24]. The recently constructed mutant library in *C. reinhardtii* provides important high-throughput methods for performing lipid metabolism research in microalgae [25]. Furthermore, ACS1 and ACS2, which are orthologs of LACS in plants, were experimentally demonstrated to be involved in lipid accumulation [1, 5, 26]. Interestingly, the *acs1* and *acs2* knock-down mutant resulted in accumulation of intracellular lipids and increased FA secretion [26]. However, overexpression of ACS2 also increased the content of intracellular lipids and starch in *C. reinhardtii* [1, 25].

As mentioned above, in a previous study, the plastid inner envelope-localized protein FAX1 was characterized to be a transporter that mediates fatty acid export from plastids in *Arabidopsis thaliana* [5, 7]. FAX proteins have seven homologs in Arabidopsis and are named FAX1-7. AtFAX1-4 is predicted to be localized in the plastid membrane and was hypothesized to be involved in lipid transport in plants. However, the fatty acid transport process from the chloroplasts of microalgae remains unknown. In the present study, we identified two novel cDNA encoding *FAX*s in *C. reinhardtii*. The constructed plasmids with CrFAXs were transformated into *C. reinhardtii* and the overexpressing strains named CrFAX1-OX and CrFAX2-OX were further characterized, and their intracellular lipid contents under normal TAP and nitrogen starvation condition were investigated, respectively. The present study is important for improving the productivity of biofuel sources from microalgae.

## Results

### Identification of potential *FAXs* genes in *C. reinhardtii*

**Fig. 1:**
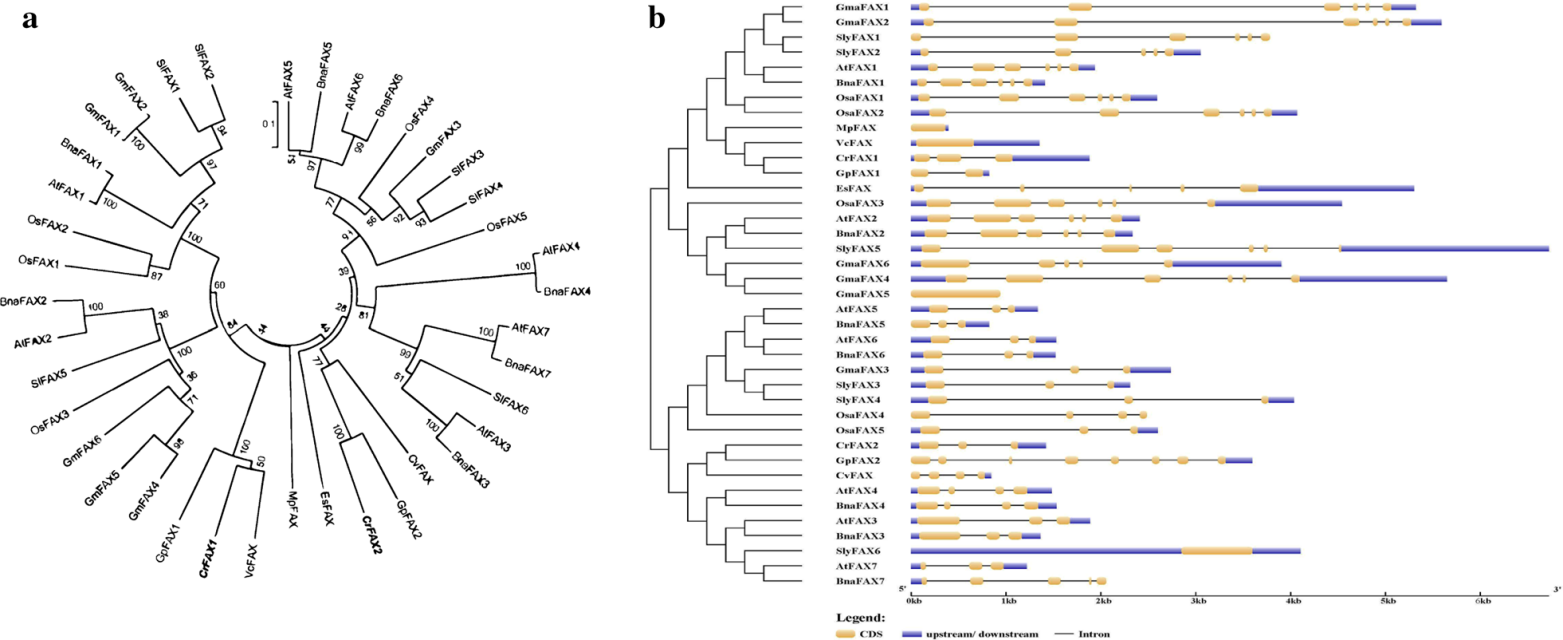
Sequence analysis of FAXs in *Chlamydomonas reinhardtii* and other algae or plant species. **a** A phylogenetic tree of FAXs was constructed using MEGA6.0. **b** Topology analysis of FAXs. At: *Arabidopsis thaliana*, Bna: *Brassica napus*, Gm: *Glycine max*, Sl: *Solanum lycopersicum*, Os: *Oryza sativa*, Cr: *Chlamydomonas reinhardtii*, Vc: *Volvox carteri*, Gp: *Gonium pectoral*, Cv: *Chlorella variabilis*, Es: *Ectocarpus siliculosus*, Mp: *Micromonas pusilla*

Genome-wide analysis was performed to predict all of the *FAX* genes in *C. reinhardtii* using genome sequence data (https://genome.jgi.doe.gov/portal/chlamy/chlamy.info.html). Initially, we identified 2 putative full-length protein sequences encoded by *FAX* genes from the *Chlamydomonas* whole genome using a BLASTP search with 7 FAXs protein sequences in Arabidopsis (collected from Phytozome v11.0) as queries. Using the primers listed in Additional file 1: Table S1 and RT-PCR, cDNA was successfully cloned. A phylogenetic tree was constructed and is shown in Fig. 1a. CrFAX1, *Volvox carteri* VcFAX and *Gonium pectoral* GpFAX1, were clustered into a single branch, while CrFAX2 and GpFAX2 were clustered into another individual branch. In addition, Arabidopsis AtFAX1, *Brassica napus* BnaFAX1 and FAX1 s from other plants were clustered in another branch (Fig. 1a; Additional file 2: Table S2). The results revealed that there may be functional diversity between CrFAX1 and CrFAX2 in *C. reinhardtii*.

The two putative *FAX* genes, *CrFAX1* (Cre10.g421750) and *CrFAX2* (Cre08.g366000), encoded proteins with lengths of 200 and 122 amino acids, respectively, and both had 3 conserved exons (Fig. 1b; Additional file 3: Figure S1). To further understand the features of the FAXs family, we predicted the putative protein motifs using the MEME program for FAX proteins in *C. reinhardtii* and 10 other species (Additional file 4: Figure S2; Additional file 5: Table S3). The numbers of motifs varied among these FAX proteins. Only motif 1 was observed to be partly conserved in both CrFAX1 and CrFAX2. In addition, both CrFAXs had two conserved motifs with AtFAX1 [7], implying that CrFAX1 and CrFAX2 may, in part, have functions similar to that of AtFAX1, but there may be functional diversity between them. To determine the structure of AtFAX1-3 and CrFAX1-2, 3D models were generated using the Phyre2 server. All of the FAXs proteins had 4 α-helix structures. The structure of CrFAXs, especially CrFAX2, was similar to AtFAX1 structure (Additional file 6: Figure S3).

### Overexpression of CrFAXs in *C. reinhardtii*

**Fig. 2:**
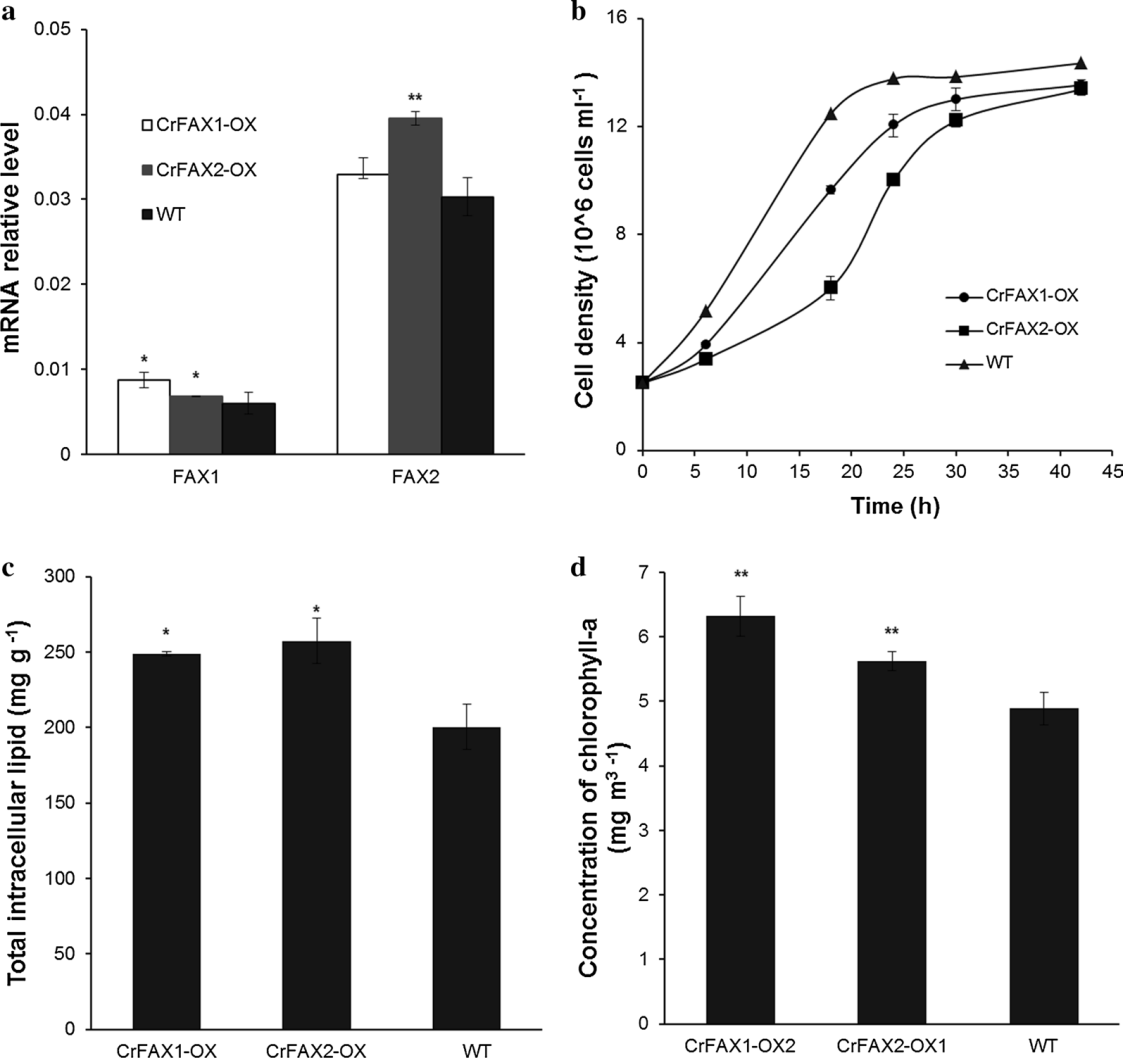
Screening and phenotype analysis of CrFAX1 and CrFAX2 overexpression transformants. **a** The expression levels of crfax1 and crfax2 in both CrFAX1 and CrFAX2 transformants (CrFAX1-OX and CrFAX2-OX), respectively, were detected by qRT-PCR assays. **b** The growth rates of the normal strain (WT) and the two CrFAXs transformants were measured according to the cell numbers. The total intracellular lipids **c** and chlorophyll a contents **d** of the normal strain and two CrFAXs transformants were detected. The results are shown as the mean expression ± standard deviation (SD) of three independent experiments. Student’s *t* test, *P < 0.05, **P < 0.01

*Chlamydomonas reinhardtii* UVM4 was transformed with the pJR38-CrFAX1 or pJR38-CrFAX2 plasmids, in which FAXs-encoding cDNAs were under the control of the *C. reinhardtii* normally used PsaD promoter (pPsaD). Successful transformants were grown under antibiotic paromomycin selective pressure, and the correct integration of the PPSaD-CrFAXs was verified with specific primers that annealed to the PPsaD promoter and *APHVIII* gene [27]. In all of the transformants studied, the *PPsaD* (380 bp) and *APHVIII* genes (360 bp) were found, confirming the correct insertion of *CrFAXs* in the genome of the microalga (Additional file 7: Figure S4). Furthermore, the overexpression lines of CrFAX1 and CrFAX2 were named CrFAX1-OX and CrFAX2-OX, respectively. qRT-PCR assays of *CrFax1* and *CrFax2* genes were performed in both lines, which confirmed that the related genes were overexpressed (Fig. 2a).

To perform preliminary screening, untransformed control (wild type, WT), CrFAX1-OX, and CrFAX2-OX strains were incubated in TAP media at the same initial cell density. The growth rate and content of neutral lipids in the strains were detected. The cell growth rate of both overexpression lines decreased 49–31% at mid-logarithmic phase and then recovered to wild-type level after 40 h of incubation (Fig. 2b). The contents of total lipids were determined by the methods described previously [1, 28]. The overexpression lines of CrFAXs consistently accumulated an 11–17% increased neutral lipid content than that of WT (Fig. 2c). Furthermore, we also detected the chlorophyll-a content of the CrFAXs overexpression lines. The results showed that both overexpression strains had an increased chlorophyll-a content compared with that of WT (Fig. 2d). Interestingly, the mutants and overexpression lines of AtFAX1 had no effect on chlorophyll contents [7]. In Arabidopsis, there should be 4 homologs of AtFAX localized in the chloroplast. The unique mutant of AtFAX may not have an effect on the chlorophyll contents. However, only two FAX members are found in *C. reinhardtii* cells, and the structure of *C. reinhardtii* is simple. Furthermore, the functions CrFAX1 and CrFAX2 may be more widespread than that of AtFAX1 in Arabidopsis.

**Fig. 3:**
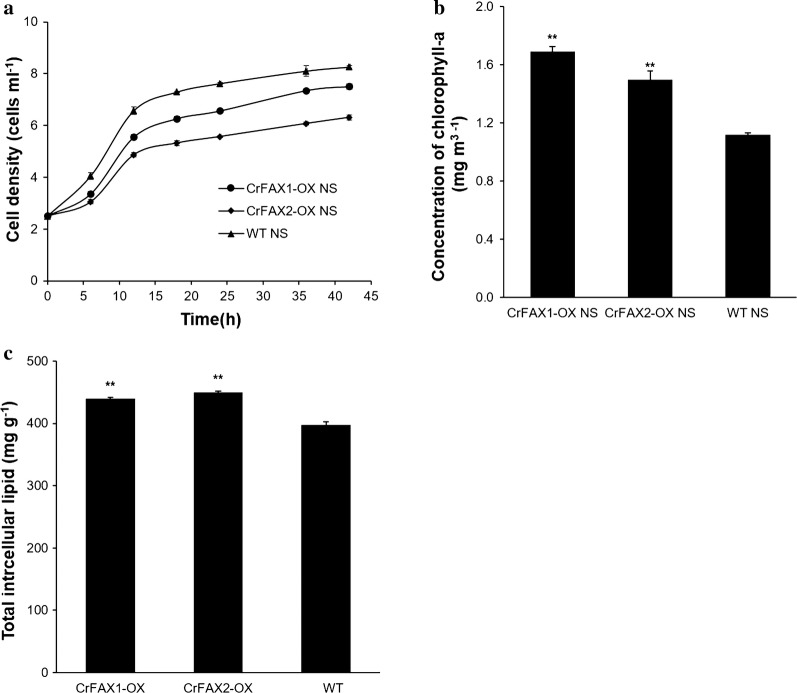
Phenotypes analysis of CrFAX1 and CrFAX2 overexpression transformants under N deprivation medium. The growth rates (**a**) concentration of chlorophyll a (**b**), and total lipid content at the middle stage (**c**) of WT and the CrFAX1 and CrFAX2 transformants were detected. The results are shown as the mean expression ± standard deviation (SD) of three independent experiments. Student’s *t* test, *P < 0.05, **P < 0.01

Microalgae prefer to form TAG when nutrient deprived. It is reported that nitrogen deprivation promotes the TAG accumulation in *C. reinhardtii* [29]. The overexpression lines of CrFAXs were further incubated in medium under nitrogen starvation (NS) conditions. The growth rates were affected in both algal lines and WT compared with the rates in TAP medium (Fig. 3a). The chlorophyll contents in overexpression lines were increased comparing with WT (Fig. 3b), which is similar to that under TAP medium. Furthermore, the lipid contents in the CrFAX1 and CrFAX2 overexpression strains were significantly increased by 17% and 15% compared with that of WT in the mid-log phase (Fig. 3c). It was shown that nitrogen starvation significantly improved lipid accumulation in both the CrFAX1 and CrFAX2 overexpression lines compared with that in WT.

### TAG accumulation in the CrFAX-OX lines

**Fig. 4:**
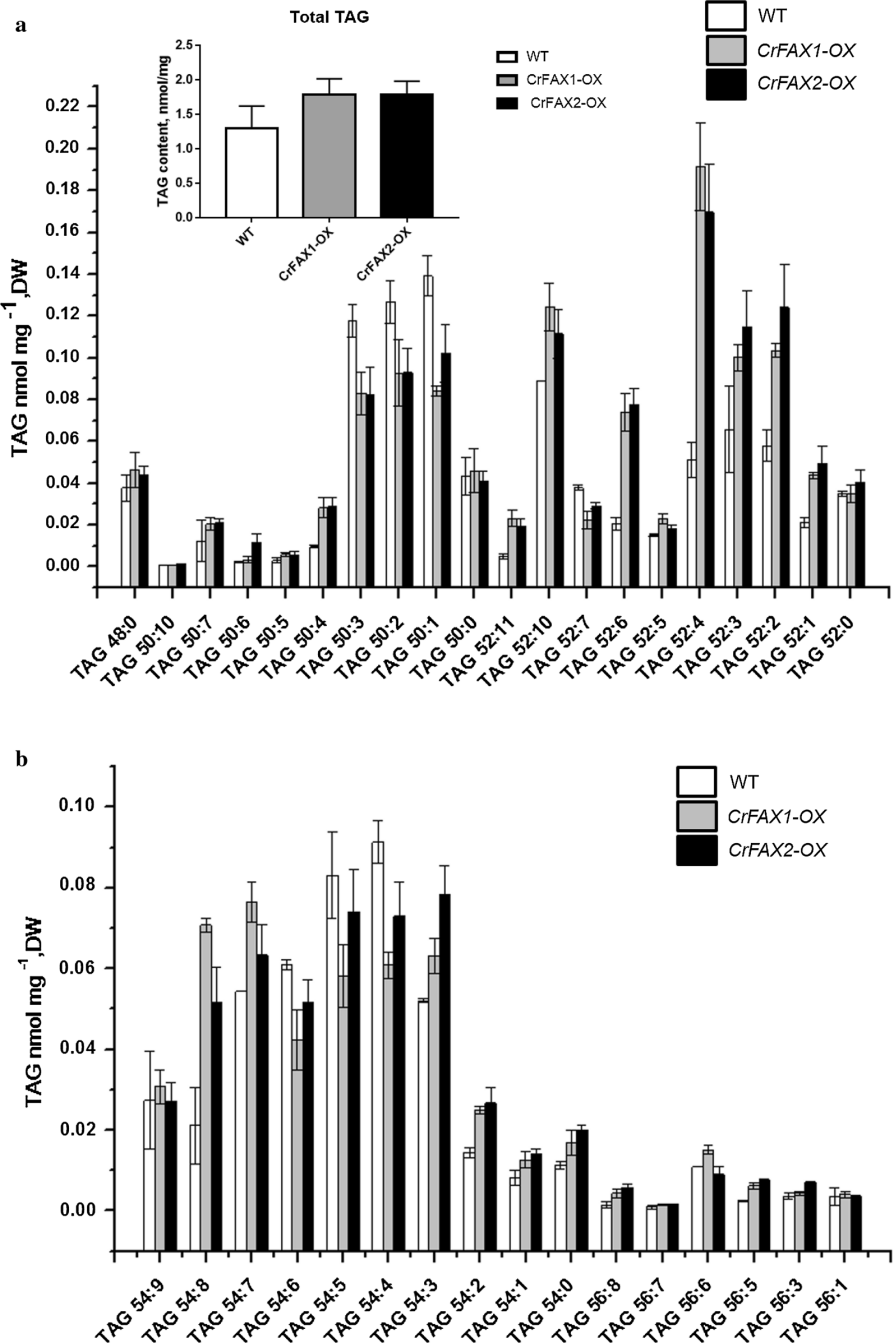
Accumulation of total TAG and the molecular species in the control and the CrFAX1 and CrFAX2 *Chlamydomonas* transformants. The total TAG content and abundance of different molecular species within TAG synthesized in the normal strain (WT) and in the CrFAX1 and CrFAX2 transformants (CrFAX1-OX and CrFAX2-OX) were determined. The results are shown as the mean expression ± standard deviation (SD) of six independent experiments. Student’s *t* test, *P < 0.05, **P < 0.01

To investigate whether CrFAX increases the content of oil droplets in algae, the TAG content was studied by HPLC–ESI–MS/MS and was shown as a molecular species within the synthesized TAG and as the total TAG (Fig. 4). Cells from the overexpression strains and WT were grown under standard conditions. The CrFAXs overexpression strains had more and larger lipid droplets than the control. The total TAG contents were significantly increased (~ 38%) in the CrFAX-OX strains compared to that in WT. Almost all of the TAG molecular species were increased in the overexpression strains (Fig. 4). These data revealed that overexpression of chloroplastic-localized CrFAXs enhanced the accumulation of TAG and confirmed that CrFAXs were effective targets to increase lipid accumulation in *C. reinhardtii* by genetic engineering.

### Characterization of the lipid species and fatty acid profiles in the CrFAX-OX lines

**Fig. 5:**
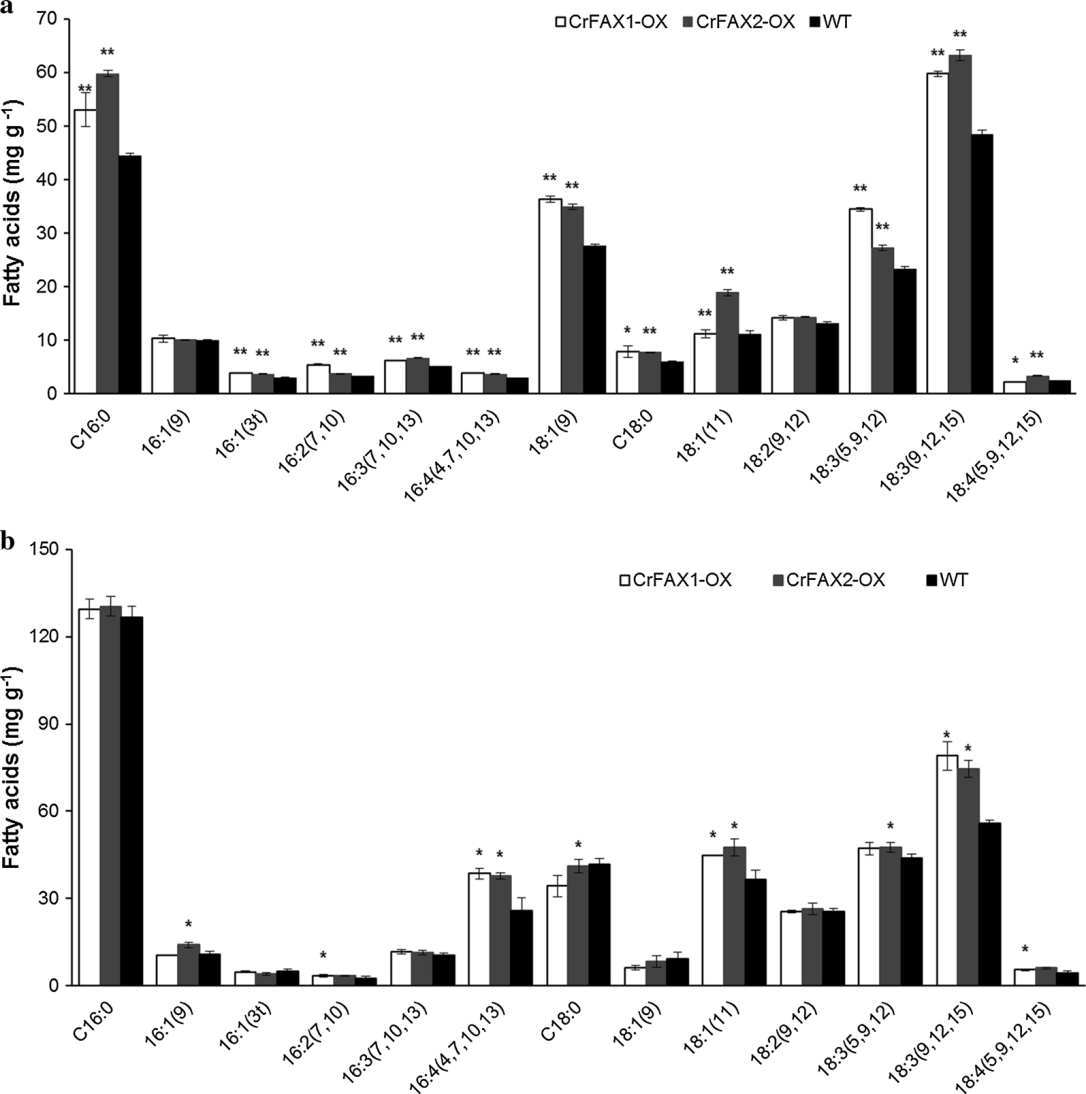
Profile of intracellular fatty acids in algae CrFAX1-OX and CrFAX2-OX and the control UVM4 in TAP or Nitrogen starvation medium. Samples from the normal strain (WT) and transformants (CrFAX1-OX and CrFAX2-OX) in TAP medium (**a**) or Nitrogen starvation medium (**b**) were analyzed, respectively. The results are shown as the mean expression ± standard deviation (SD) of three independent experiments. Student’s *t* test, *P < 0.05, **P < 0.01

To further characterize lipid biosynthesis in the CrFAXs overexpression strains, the total lipid content and fatty acids contents were detected by a gas chromatograph (GC-FID). The fatty acids composition analysis results showed that almost all compositions increased, with a significant difference (*P* < 0.01), compared with WT. Although, major lipids in both overexpression strains were still C18:3n, C18:1 and C16:0, and their content showed an obvious increase (Fig. 5a). These results indicated that CrFAXs may be involved in the transport of both saturated and unsaturated short-chain fatty acids, such as C18:3n, C18:1 and C16:0.

Under nitrogen starvation (NS) conditions, the fatty acid compositions in both CrFAXs overexpression strains were also increased in comparison with those in WT (Fig. 5b). C16:4 (4,7,10,13) and C18:1 (11) showed obvious upregulation, and the C18:3 (9,12,15) contents in the CrFAXs-OX lines were increased by 62% and 69% compared to that of WT. The result revealed that CrFAXs can be used to increase the unsaturated fatty acid C18:3 (9,12,15) content under nitrogen starvation conditions.

**Fig. 6:**
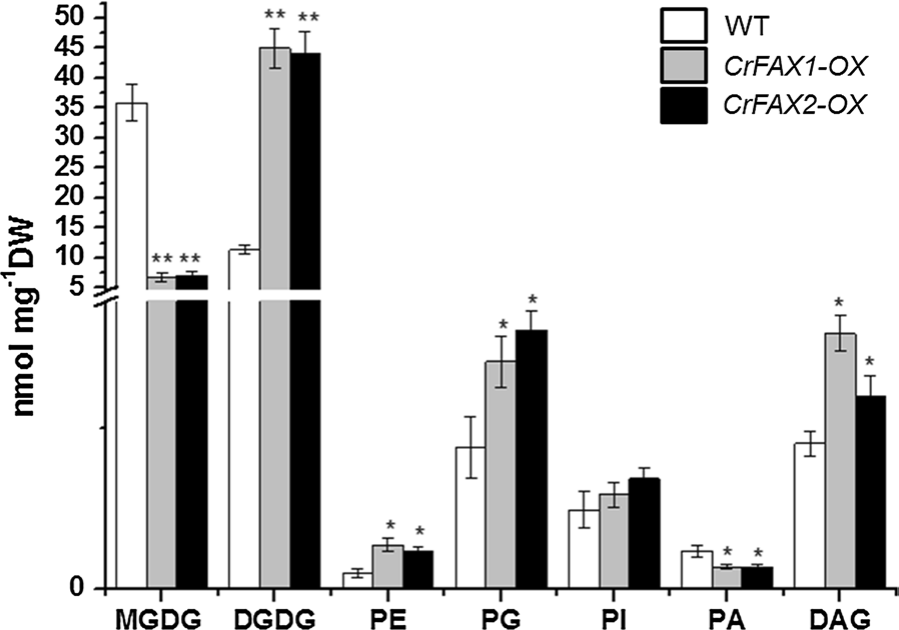
Accumulation of the major polar membrane lipids species in the control and transformants. The major lipid classes in glycolipid and phospholipid extracts from the normal strain (WT) and transformants (CrFAX1-OX and CrFAX2-OX) were identified by UPLC–MS/MS analysis. Abbreviations: monogalactosyl diacylglycerol (MGDG), digalactosyl diacylglycerol (DGDG), phosphatidyl glycerol (PG), sulfolipid sulfoquinovosyl diacylglycerol (SQDG), diacylglycerol trimethyl homoserine (DGTS), phosphatidylethanolamine (PE). The results are shown as the mean expression ± standard deviation (SD) of six independent experiments. Student’s *t* test, *P < 0.05, **P < 0.01

The quantification of the major polar lipids was also investigated, and the results revealed that the main lipid component of the thylakoid membrane, MGDG, was decreased in the CrFAXs overexpression lines, which is in agreement with previous reports in Arabidopsis [7]. The decrease of MGDG is particularly noteworthy, which was reduced to 19% of that of WT. However, the other abundant lipids in plastid and thylakoid membranes, Diacylglycerol (DAG), digalactosyl diacylglycerol (DGDG), phosphatidylglycerol (PG) and phosphatidylethanolamine (PE), increased in the CrFAX-OX lines (Fig. 6). Furthermore, the betaine lipid, diacylglycerol trimethyl homoserine (DGTS), which is the major extraplastidic lipid in *C. reinhardtii* and replaced phosphatidylcholine (PC), was also increased in the CrFAX overexpression strains.

### Transcriptomic analysis and Differential expression levels of chloroplastic acetyl-CoA synthetase in the CrFAX-OX lines

To further investigate the characteristics of CrFAXs in lipid biosynthesis in microalgae, transcriptomic analysis was performed using RNA-sequencing. Six cDNA libraries, two replicates of WT, CrFAX1-OX, and CrFAX2-OX, were generated for further analysis. A total of approximately 23.4–23.9 million raw reads were subsequently obtained. We ultimately obtained 22.6–23.3 million clean reads from each cDNA library. More than 77% of the clean reads were mapped to the reference genome for all samples (Additional file 8: Table S4). In addition, more than 90% of the clean reads were uniquely mapped to all of RNA-Seq samples (Table 1; Additional file 9: Table S5). When comparing the transcriptome data between CrFAXs-OX and WT, we found that 303 and 182 genes were upregulated, and 104 and 124 genes were downregulated, respectively. To better understand the function of these differentially expressed genes (DEGs), we preformed Gene Ontology (GO) and Kyoto Encyclopedia of Genes and Genomes (KEGG) metabolic pathway analyses. Within the “biological process” category of the GO analysis, there were 301 and 256 regulated genes, respectively. In the “cellular component” category, there were 422 and 350 regulated genes. In the “molecular function” category, 218 and 172 genes were regulated (Additional file 10: Figure S5). KEGG metabolic pathway analysis revealed that 71 DEGs were distributed among 15 and 17 metabolic pathways for the regulated genes, respectively (Additional file 11: Figure S6). According to the function annotation of the DEGs, a total of 6 genes involved in lipid metabolism are listed in Additional file 9: Table S5. The regulated expression of chloroplastic acetyl-CoA synthetases, *ACS1* and *ACS2* is also shown in the list.

**Table 1:** Summary of sequencing data for each sample

Sample	Raw data size (bp)	Raw reads number	Clean data size (bp)	Clean reads number	Clean data rate (%)
CrFAX1ox1	1,170,512,600	23,410,252	1,151,440,450	23,028,809	98.37
CrFAX1ox2	1,188,013,300	23,760,266	1,130,519,650	22,610,393	95.16
CrFAX2ox1	1,163,204,700	23,264,094	1,132,009,700	22,640,194	97.31
CrFAX2ox2	1,165,555,450	23,311,109	1,150,703,250	23,014,065	98.72
WT1	1,195,937,400	23,918,748	1,134,751,300	22,695,026	94.88
WT2	1,173,795,650	23,475,913	1,163,434,850	23,268,697	99.11

**Fig. 7:**
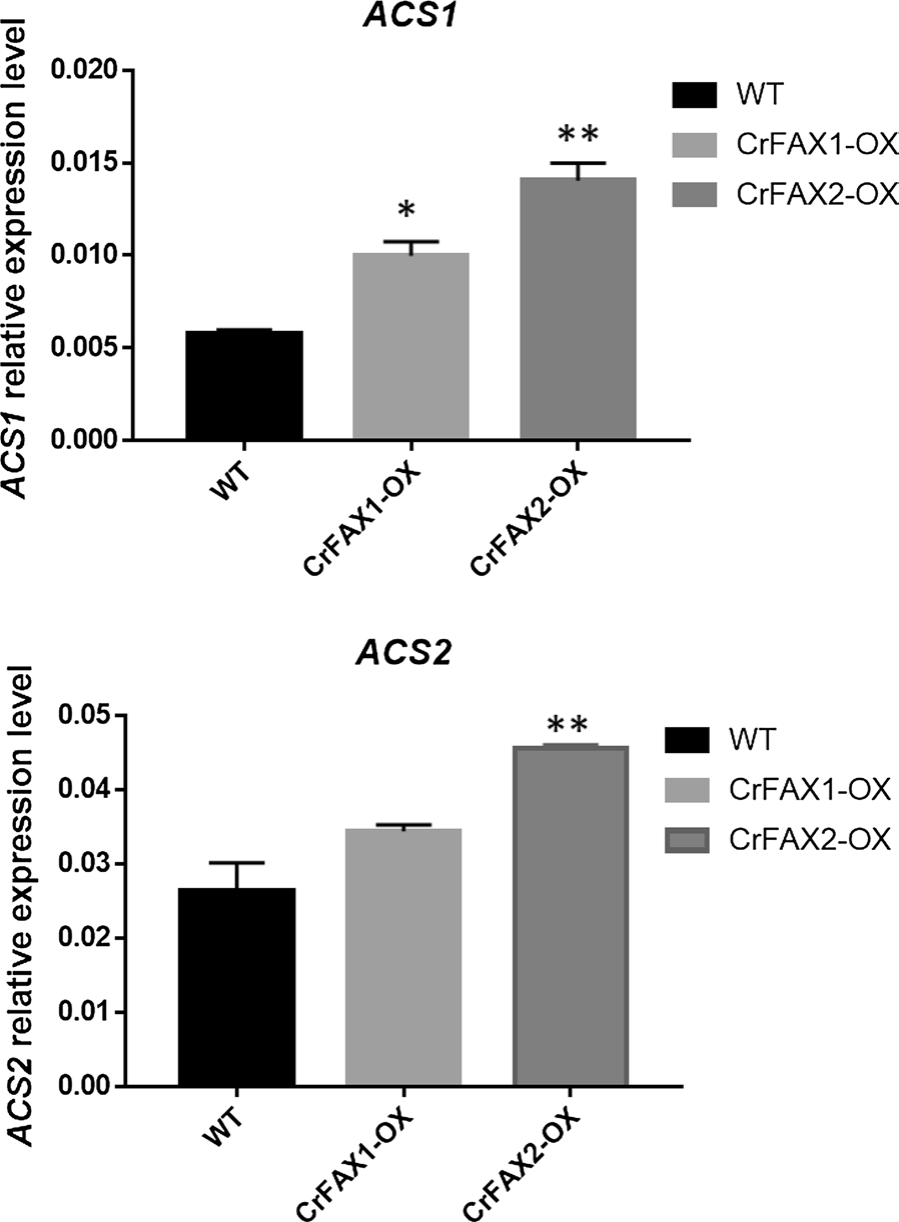
Relative expression of gene *ACSs* in CrFAX1-OX and CrFAX2-OX. The relative expression levels of CrACS1 and CrACS2 in CrFAXs overexpression lines and WT were detected by qRT-PCR analysis. The results are shown as the mean expression ± standard deviation (SD) of three independent experiments. Student’s *t* test, *P < 0.05, **P < 0.01

We further analyzed the transcription levels of *ACS1* and *ACS2*, which were reported to be involved in acetyl-CoA synthesis and further TAG accumulation [1, 26]. The expression profile of these two genes in the CrFAX-OX strains and WT was analyzed by real-time quantitative PCR (Fig. 7). The result showed that both *ACS1* and *ACS2* were upregulated in the two CrFAX-OX strains compared to WT. The expression of *ACS1* increased by almost 2.5 times in the CrFAX2-OX strain and the expression of *ACS2* was upregulated by approximately 1.9 times in the CrFAX2-OX strain compared to WT (Fig. 7).

## Discussion

### Characterization of CrFAXs in *C. reinhardtii*

In the present study, two *FAX* genes were identified in the *C. reinhardtii* genome (Fig. 1 and Additional file 3: Figure S1, Additional file 4: Figure S2, Additional file 5: Figure S3). Each gene had two conserved motifs with *AtFAX1* in Arabidopsis. The lengths of the CrFAXs were 200 and 122 amino acids, which were similar to AtFAX1 [7]. Further, these two *FAX* genes displayed a marked variation in gene structure and protein motifs, implying a high degree of functional complexity in *C. reinhardtii*. Furthermore, both CrFAXs had at least 3 α-helix structures, similar to AtFAX1. Phylogenetic tree analysis revealed that CrFAX1 and CrFAX2 might have functional diversity with FAXs in plants. Sequence analysis of CrFAX1 and CrFAX2 implied that both CrFAXs might have similar but different functions with AtFAX1.

### Function of CrFAXs involved in fatty acids transport

In a previous study, fatty acids C16:0, C18:0, C18:1 and C18:3 were shown to be substrates for transport by AtFAX1 when heterologously expressed in yeast cells [7]. In *C. reinhardtii*, CrLACSs can transport C16:0, C18:0, C18:1, C18:2 and C18:3 according to a functional analysis experiment in yeast strains [26]. To determine whether CrFAXs are involved in fatty acid transport, *Crfax1* and *Crfax2* cDNA was separately cloned into the yeast expression vector pYES2 and then electroporated into the yeast (*S. cerevisiae*) strain BY4741. C16:0, C18:0, C18:1, C18:2 and C18:3 were used as carbon sources in the medium, and both CrFAXs affected fatty acid uptake into yeast (Additional file 12: Figure S7). In comparison, CrFAX1 prefers C16:0, C18:0, and C18:2, and CrFAX2 prefers to be involved in C16:0, C18:1, C18:2 and C18:3 metabolism (Additional file 12: Figure S7). Although further yeast complementation research with yeast mutant *fat1p* is necessary to clarify the potential transport function of CrFAXs, the present results showed that both CrFAXs may perform similar functions to AtFAX1.

### The changed lipid contents and lipid compositions in the overexpression transformants of CrFAXs

In Arabidopsis, AtFAX1 mainly affects the contents of C16:0, C18:2, C20:0, C24:0 and C26:0. However, in the present study, CrFAXs overexpression accelerated both saturated and unsaturated short-chain fatty acids, such as C18:3n, C18:1 and C16:0 (Fig. 5). And the TAG compositions, such as TAG52:4, TAG52:3, TAG52:2, TAG54:8, which are composed by both saturated and unsaturated fatty acids, were significantly upregulated in the CrFAX-OX strains comparing with WT (Fig. 4). The results reveal that CrFAXs may be involved in both saturated and unsaturated fatty acid, especially C18:3n, C18:1 and C16:0, transport processes. Compared with the fatty acid composition changes in TAP medium, the C16:4 and C18:3 contents in the CrFAX-OX strains were significantly increased under NS conditions [1]. This result revealed that CrFAXs may not only be involved in fatty acid export for TAG biosynthesis (Fig. 4) but also improve the fatty acid supply for thylakoid membrane lipid synthesis, which is important for photosynthesis in algae [15].

The overexpression of both CrFAXs in *C. reinhardtii* increased the chlorophyll contents no matter in TAP medium or in nitrogen deprivation conditions (Figs. 2, 3). The knockout or overexpression of AtFAX1 showed no chlorophyll differences in Arabidopsis [5, 7]. The chloroplast of *C. reinhardtii,* which occupy 70% of unique cells, is the main organ for photosynthesis. The effect of the fatty acid distribution in chloroplasts may severely impact photosynthesis metabolism in *C. reinhardtii* [1]. CrFAXs overexpression transformants accumulated more DGDG, which is the main membrane component of thylakoids in *C. reinhardtii*. Further, overexpression of CrFAXs accelerated the utilization of C16 and C18 fatty acids in the chloroplast, which may provide a positive signal to the photosynthesis system.

According to the results in the present study, we made a hypothesis for the potential function of both CrFAX1 and CrFAX2 in *C. reinhardtii*: CrFAX1 and CrFAX2 should be localized at the chloroplast envelope to be involved in mediating the C16 and C18 fatty acids export into cytosol. The exported fatty acids are further transferred to be acyl-CoA by ACS. And the acyl-CoA is imported into ER for TAG biosynthesis. When CrFAX1 and CrFAX2 were overexpressed in *C. reinhardtii*, more chloroplast-localized C16 and C18 fatty acids were exported into cytosol, and more acyl-CoA was synthesized. And furthermore, more acyl-CoA was used for TAG biosynthesis.

## Conclusion

In the present study, two FAXs cDNAs, *CrFax1* and *CrFax2*, in *C. reinhardtii* were successfully cloned and characterized. Both proteins were involved in mediating fatty acid export from chloroplast into the cytosol for Acyl-CoA synthesis. Overexpression of either *CrFax1* or *CrFax2* in *C. reinhardtii* resulted in intracellular TAG accumulation. These results confirmed a novel strategy for increasing the microalgae oil contents. According to our present results, microalgal oil may also be able to be increased in other algae species by this method, which may promote the industrial application of microalgal biofuels in the future.

## Methods

### Strains, vectors and culture condition

*Chlamydomonas reinhardtii* cc503 (cell wall-deficient strain) was obtained from the Chlamydomonas Resource Center. *C. reinhardtii* UVM4 (cell wall-deficient strain) and the vector pJR38 were kindly provided by Professor Ralph Bock, Max-Planck Institute for Molecular Plant Physiology, Germany [27]. pJR38 was used for gene expression in *C. reinhardtii*. The vector pYES2.0 (Invitrogen, USA) with the GAL1 promoter was used for FAXs gene expression in *S. cerevisiae* (BY4741).

*C. reinhardtii* was cultured in Tris–acetate-phosphate (TAP) medium (30-mL medium in the 200-mL triangular flask) under continuous light (60 μmol m^−2^ s^−1^ at 25 °C and 100 rpm). The *C. reinhardtii* cells at the mid-logarithmic phase of growth (OD_660_ = 0.8) were harvested by centrifugation, washed and resuspended in TAP or Nitrogen starvation medium (TAP-N medium; KCl substituted for NH_4_Cl). The cells were incubated in TAP or Nitrogen starvation medium with the beginning cell density at 2.5 × 10^6^ mL^−1^ under continuous light (60 μmol m^−2^ s^−1^ at 25 °C and 100 rpm).

### Cloning of FAXs genes and sequence analysis

The putative FAXs genes in *C. reinhardtii* were identified from the NCBI genome database using the nucleotide sequence of *A. thaliana* by BLAST. Total RNA was isolated by EZ-10 DNAaway RNA Mini-preps Kits (BBI Life Sciences) according to the manufacturer’s instructions. The first strand of cDNA was synthesized using oligo-dT as the reverse primer and Reverse Transcriptase M-MLV according to the PrimeScript RT reagent kit with gDNA Eraser (Takara). All of the primers were designed according to the BLAST results using Vector NTI software. In addition, *CrFax* cDNA was amplified using premix Ex Taq™ version 2.0 (Takara). The PCR products were then cloned into the pGEM-T vector after purification and were sequenced. The protein sequences were aligned using Vector NTI, and a phylogenetic tree was constructed using MEGA 6.0; topology analysis was performed using Phyre2 server software.

### Plasmid construction and transformation

cDNAs of CrFaxs were inserted into the overexpression vector pJR38 as described by Neupert et al. [27] and Sizova et al. [30]. Transformation of the constructed plasmids CrFax1-pJR38 and CrFax2-pJR38 into *C. reinhardtii* was performed by the glass beads agitation method according to Kindle [31]. Paromomycin (10 μg mL^−1^) was used for antibiotic selection of the transformants. To verify the successful transformation of CrFaxs in *C. reinhardtii*, genomic DNA from the transformants was extracted and used as templates for PCR amplification of APHVIII and PPsaD in the pJR38 vector according to the description in Neupert et al. [27].

### qRT-PCR analysis

Total RNA was extracted and cDNA was synthesized as described above. Using SYBR Premix ExTaq Kits (Takara), 2 μL of cDNA was used for qRT-PCR according to the manufacturer’s instruction on an ABI Prism 7900 Sequence Detection system. The primers used for qRT-PCR are listed in Additional file 1: Table S1, and the housekeeping gene GBIP (G protein subunit-like protein) was used as the Ref. [32]. At least three biological replicates were performed for each treatment.

### Chlorophyll a content measurement

The chlorophyll a content was determined according to Mera et al. [33] with some modifications. Microalgal cells were harvested and suspended in 95% (v/v) alcohol. The suspension was kept at 4 °C in the dark for 10 min and centrifuged to remove cell debris. The absorbance of the supernatant at 649 and 665 nm was measured in an UV–Vis spectrophotometer (DU730, Beckman, Germany). A 95% alcohol solution was used as the blank. The concentration (mg/mL) of Chl-a was calculated with the following equation: Chl-a (mg/l) = [(A_665_–A_750_) × 13.7 − (A_649_–A_750_) × 5.76] × 2.5. At least three biological replicates were performed for each treatment.

### Total lipid extraction

Total lipid was extracted using a method described previously [1, 28]. Algal cells cultured to the logarithmic phase were centrifuged to remove the supernatant and freeze-dried using a vacuum freeze dryer (Telstar lyoQuest-55 Plus). Algal cells were rehydrated with 1 mL of methanol and 2 mL of a 2% (m/v) NaOH solution and vortexed for 2 min on a vortex shaker. The mixed solution was placed in a 60 °C water bath for 1 h. After cooling to room temperature, 2 mL of chloroform was added and the solution was agitated again for 2 min. After centrifugation of the mixed solution, the lower layer was transferred to a dry test tube, and 1 mL of *n*-hexane was added to the remaining upper layer and vortexed again for 2 min. After centrifugation, the solution again appeared to be delaminated. The upper solution (*n*-hexane) was mixed with the just-transferred chloroform solution. The mixture was dried under high-purity N_2_, and total lipids were extracted for measurement. At least three biological replicates were performed for each treatment.

### GC-FID analysis of fatty acid methyl esters

Collected algal cell pellets were dried by vacuum freezing for 48 h. Dry cell pellets (5 mg) were re-suspended in 2 mL of a 2% (m/v) sodium hydroxide methanol solution and incubated at 80 °C for 1 h. Then, 2 mL of a 5% (m/v) sulfuric acid methanol solution was added to the solution and incubated at 80 °C for 1 h. Next, 1 mL of NaCl buffer and 1 mL of *n*-hexane were added into the solution and were re-suspended. After 3000*g* centrifugation for 2 min, 150 μL of the supernatant was used for gas chromatography flame ionization detection (GC-FID) analysis using an Agilent 7890A gas chromatograph fitted with an Agilent DB-23 (60 m × 0.25 mm × 0.25 μm) column as described previously [1]. A 1 μl aliquot of each sample was analyzed with a 1:10 split injection and constant flow rate of 1.5 mL min^−1^. The oven temperature cycle was initially held at 130 °C for 1 min, then increased to 220 °C at a rate of 1.5 °C min^−1^ for 3 min, and finally increased to 230 °C at a rate of 40 °C min^−1^. The temperature was then held for 3 min, for a total run time of 17 min per sample. Analysis was carried out using Agilent Chemstation software. The retention time and identity of each peak were calibrated using FAME 37 Component FAME mix (Supelco) and methylated Qualmix Menhaden oil (Larodan) and quantified using pentadecanoic acid (0.4 mg mL^−1^) as an internal standard. At least three biological replicates were performed for each treatment.

### Quantitative lipid analysis

Lipids were extracted according to the method of Welti et al. [34]. Phospholipids and galactolipids were analyzed by a shotgun lipidomics method described by Welti et al. [34] using a Triple Quad 4000 LC–MS/MS [34]. Triacylglycerols (TAGs) were quantitatively analyzed using a reverse-phase HPLC–ESI–MS/MS approach described previously [35]. Neutral loss scans of fatty acids were used to determine the species of TAG and DAG. The lipids in each class were quantified by comparison with the internal standard of the same class. Lipid standards were purchased from Avanti Polar Lipids, Inc. (Alabaster, AL, USA), and the Lipid Metabolites and Pathways Strategy (LIPID MAPS, USA).

### Transcriptome sequencing and data analysis

The total RNA was extracted from the *C. reinhardtii* strains of CrFAXs-OX and WT at mid-log stage. Sequencing was performed using an Illumina HiSeq™ 2000 sequencing platform. At least two biological replicates were used for each strain species. The gene expression levels were analyzed by HTSeq software, and the model was a union, according to the way as Li et al. [36]. The FPKM was consisted as the estimation method to evaluate the effect of sequencing depth and gene length on the number of counts. The readcount data obtained from the analysis of the gene expression levels were normalized by TMM and analyzed by DEGseq software. The standard for selection of the biologically repeated data was padj < 0.05.

### Statistical analysis

All data were statistically analyzed using SPSS 18.0 and GraphPda Prism 5. Analysis of variance was performed on data sets, with the mean and SD of each treatment calculated. Multiple comparisons with Duncan’s test or Student’s *t* test were mainly used to compare the mean values between strains (*P < 0.05; **P < 0.01).

## Additional files

**Additional file 1: Table S1.** Complete list of primers used in this study.

**Additional file 2: Table S2.** All gene names and accession numbers presented in this study.

**Additional file 3: Figure S1.** The protein sequences Alignment of CrFAX1, CrFAX2 and AtFAX1.

**Additional file 4: Figure S2.** The conserved motifs prediction was constructed using MEME.

**Additional file 5: Table S3.** The information of conserved motifs.

**Additional file 6: Figure S3.** 3D modeling prediction of CrFAXs and AtFAXs.

**Additional file 7: Figure S4.** Screening mutants by using PCR amplification.

**Additional file 8: Table S4.** QC items for each sample.

**Additional file 9: Table S5.** The lipid metabolism related genes regulated in CrFAXs overexpression strains.

**Additional file 10: Figure S5.** Distribution of most abundant Gene ontology (GO) terms assigned to the CrFAX1 vs WT (a) and CrFAX2 vs WT (b).

**Additional file 11: Figure S6.** KEGG classification on DEGs for CrFAX1 vs WT (a) and CrFAX2 vs WT (b).

**Additional file 12: Figure S7.** Substrate utilization profiles of yeast strains expressing *crfax1* and *crfax2.*

## Abbreviations

ACSs: long-chain acyl-CoA synthetases
FAs: fatty acids
qRT-PCR: quantitative reverse transcription-PCR
TAG: triacylglycerols
PE: phosphatidylethanolamine
PG: phosphatidylglycerol
DGDG: the galactolipid digalactosyl diacylglycerol
MGDG: monogalactosyldiacylglycerol
DGTS: the betaine lipid diacylglycerol trimethyl homoserine
GC-FID: gas chromatography flame ionization detection
GBIP: G protein subunit-like protein
FAX: fatty acid exporter

## References

[1] Rengel R, Smith RT, Haslam RP, Sayanova O, Vila M, León R (2018). Overexpression of acetyl-CoA synthetase (ACS) enhances the biosynthesis of neutral lipids and starch in the green microalga *Chlamydomonas reinhardtii*. Algal Res.

[2] Banerjee C, Singh PK, Shukla P (2016). Microalgal bioengineering for sustainable energy development: recent transgenesis and metabolic engineering strategies. Biotechnol J.

[3] Wang C, Chen X, Li H, Wang J, Hu Z (2017). Artificial miRNA inhibition of phosphoenolpyruvate carboxylase increases fatty acid production in a green microalga *Chlamydomonas reinhardtii*. Biotechnol Biofuels.

[4] Li-Beisson Y, Shorrosh B, Beisson F, Andersson MX, Arondel V, Bates PD, Baud S, Bird D, DeBono A, Durrett TP, Franke RB, Graham IA, Katayama K, Kelly AA, Larson T, Markham JE, Miquel M, Molina I, Nishida I, Rowland O, Samuels L, Schmid KM, Wada H, Welti R, Xu C, Zallot R, Ohlrogge J (2013). Acyl-lipid metabolism. Arabidopsis Book.

[5] Li N, Xu C, Li-Beisson Y, Philippar K (2016). Fatty acid and lipid transport in plant cells. Trends Plant Sci.

[6] Penfield S, Li Y, Gilday AD, Graham S, Graham IA (2006). Arabidopsis ABA INSENSITIVE4 regulates lipid mobilization in the embryo and reveals repression of seed germination by the endosperm. Plant Cell.

[7] Li N, Gugel IL, Giavalisco P, Zeisler V, Schreiber L, Soll J, Philippar K (2015). FAX1, a novel membrane protein mediating plastid fatty acid export. PLoS Biol.

[8] Koo AJ, Ohlrogge JB, Pollard M (2004). On the export of fatty acids from the chloroplast. J Biol Chem.

[9] Schnurr JA, Shockey JM, de Boer GJ, Browse JA (2002). Fatty acid export from the chloroplast. Molecular characterization of a major plastidial acyl-coenzyme A synthetase from Arabidopsis. Plant Physiol.

[10] Kim S, Yamaoka Y, Ono H, Kim H, Shim D, Maeshima M, Martinoia E, Cahoon EB, Nishida I, Lee Y (2013). AtABCA9 transporter supplies fatty acids for lipid synthesis to the endoplasmic reticulum. Proc Natl Acad Sci USA.

[11] Fan J, Yan C, Roston R, Shanklin J, Xu C (2014). Arabidopsis lipins, PDAT1 acyltransferase, and SDP1 triacylglycerol lipase synergistically direct fatty acids toward beta-oxidation, thereby maintaining membrane lipid homeostasis. Plant Cell.

[12] Fulda M, Schnurr J, Abbadi A, Heinz E, Browse J (2004). Peroxisomal acyl-CoA synthetase activity is essential for seedling development in *Arabidopsis thaliana*. Plant Cell.

[13] Kunz HH, Scharnewski M, Feussner K, Feussner I, Flugge UI, Fulda M, Gierth M (2009). The ABC transporter PXA1 and peroxisomal-oxidation are vital for metabolism in mature leaves of Arabidopsis during extended darkness. Plant Cell.

[14] Kim Y, Terng EL, Riekhof WR, Cahoon EB, Cerutti H (2018). Endoplasmic reticulum acyltransferase with prokaryotic substrate preference contributes to triacylglycerol assembly in *Chlamydomonas*. Proc Natl Acad Sci USA.

[15] Kong F, Romero IT, Warakanont J, Li-Beisson Y (2018). Lipid catabolism in microalgae. New Phytol..

[16] Li-Beisson Y, Beisson F, Riekhof W (2015). Metabolism of acyl-lipids in *Chlamydomonas reinhardtii*. Plant J.

[17] Liu J, Han D, Yoon K, Hu Q, Li Y (2016). Characterization of type 2 diacylglycerol acyltransferases in *Chlamydomonas reinhardtii* reveals their distinct substrate specificities and functions in triacylglycerol biosynthesis. Plant J.

[18] Warakanont J, Tsai CH, Michel EJ, Murphy GR, Hsueh PY, Roston RL, Sears BB, Benning C (2015). Chloroplast lipid transfer processes in *Chlamydomonas reinhardtii* involving a TRIGALACTOSYLDIACYLGLYCEROL 2 (TGD2) orthologue. Plant J.

[19] Fan J, Andre C, Xu C (2011). A chloroplast pathway for the de novo biosynthesis of triacylglycerol in *Chlamydomonas reinhardtii*. FEBS Lett.

[20] Fulda M, Shockey J, Werber M, Wolter FP, Heinz E (2002). Two long-chain acyl-CoA synthetases from *Arabidopsis thaliana* involved in peroxisomal fatty acid β-oxidation. Plant J.

[21] Jessen D, Roth C, Wiermer M, Fulda M (2015). Two activities of long-chain acyl-coenzyme A synthetase are involved in lipid trafficking between the endoplasmic reticulum and the plastid in Arabidopsis. Plant Physiol.

[22] Lu B, Xu C, Awai K, Jones AD, Benning C (2007). A small ATPase protein of Arabidopsis, TGD3, involved in chloroplast lipid import. J Biol Chem.

[23] Xu C, Fan J, Froehlich JE, Awai K, Benning C (2005). Mutation of the TGD1 chloroplast envelope protein affects phosphatidate metabolism in Arabidopsis. Plant Cell.

[24] Xu C, Fan J, Riekhof W, Froehlich JE, Benning C (2003). A permease-like protein involved in ER to thylakoid lipid transfer in Arabidopsis. EMBO J.

[25] Li X, Zhang R, Patena W, Gang SS, Blum SR, Ivanova N, Yue R, Robertson JM, Lefebvre PA, Fitz-Gibbon ST, Grossman AR, Jonikas MC (2016). An indexed, mapped mutant library enables reverse genetics studies of biological processes in *Chlamydomonas reinhardtii*. Plant Cell.

[26] Jia B, Song Y, Wu M, Lin B, Xiao K, Hu Z, Huang Y (2016). Characterization of long-chain acyl-CoA synthetases which stimulate secretion of fatty acids in green algae *Chlamydomonas reinhardtii*. Biotechnol Biofuels.

[27] Neupert J, Karcher D, Bock R (2009). Generation of Chlamydomonas strains that efficiently express nuclear transgenes. Plant J.

[28] Ma Y, Wang Z, Zhu M, Yu C, Cao Y, Zhang D, Zhou G (2013). Increased lipid productivity and TAG content in *Nannochloropsis* by heavy-ion irradiation mutagenesis. Bioresour Technol.

[29] Miller R (2010). Changes in transcript abundance in Chlamydomonas reinhardtii following nitrogen deprivation predict diversion of metabolism. Plant Physiol.

[30] Sizova I, Fuhrmann M, Hegemann P (2002). A streptomyces rimosus aphVIII gene coding for a new type phosphotransferase provides stable antibiotic resistance to *Chlamydomonas reinhardtii*. Gene.

[31] Kindle K (1990). High-frequency nuclear transformation of *Chlamydomonas reinhardtii*. Proc Natl Acad Sci USA..

[32] Maruyama S, Tokutsu R, Minagawa J (2014). Transcriptional regulation of the stress-responsive light harvesting complex genes in *Chlamydomonas reinhardtii*. Plant Cell Physiol.

[33] Mera R, Torres E, Abalde J (2016). Effects of sodium sulfate on the freshwater microalga *Chlamydomonas moewusii*: implications for the optimization of algal culture media. J Phycol.

[34] Welti R, Li W, Li M, Sang Y, Biesiada H, Zhou HE, Rajashekar CB, Williams TD, Wang X (2002). Profiling membrane lipids in plant stress responses. Role of phospholipase D alpha in freezing-induced lipid changes in Arabidopsis. J Biol Chem.

[35] Lam SM, Tong L, Duan X, Petznick A, Wenk MR, Shui G (2014). Extensive characterization of human tear fluid collected using different techniques unravels the presence of novel lipid amphiphiles. J Lipid Res.

[36] Li N (2018). Genome-wide analysis and expression profiling of the HMA gene family in Brassicanapus under cd stress. Plant Soil.

