# Supplementary material for: Characterization of Fatty Acid Exporters involved in fatty acid transport for oil accumulation in the green alga Chlamydomonas reinhardtii
Source: Biotechnol Biofuels. 2019 Jan 12;12:14. doi: 10.1186/s13068-018-1332-4 (PMC6330502; doi:10.1186/s13068-018-1332-4)
Supplement: Supplementary file 1 — Additional file 1: Table S1. Complete list of primers used in this study. [file 13068_2018_1332_MOESM1_ESM.docx]

**Additional file 1: Table S1 Complete list of primers used in this study**

| **Primer Name** | **Primer Sequence(5’-3’)** | **Description** |
| --- | --- | --- |
| CrFAX1F NdeI | CATATGGCTGCTTCCCTGCTGCA | Primers used for construction of overexpression plasmid pJR38-CrFAX1 |
| CrFAX1R HA EcoRI | GAATTCTTAGGCGTAGTCCGGCACGTCGTACGGGTACTCGGCCTTGCCGGCG |  |
| CrFAX2F NdeI | CATATGTATGACTTTTGCTTTTCGCCC | Primers used for construction of overexpression plasmid pJR38-CrFAX2 |
| CrFAX2R HA EcoRI | GAATTCTTAGGCGTAGTCCGGCACGTCGTACGGGTAGTGCGCCTTGCCGTGC |  |
| APFAXF | GGAGGATCTGGACGAGGAGCGGAAG | Primers used for mutants screening for APHVIII of vector pJR38 in transgenetic algae |
| APFAXR | CCTCAGAAGAACTCGTCCAACAGCC |  |
| PPFAXF | GTAAAACGACGGCCAGT | Primers used for mutants screening for PPsaD of vector pJR38 in transgenetic algae |
| PPFAXR | CGAGCCCTTCGAACAGCCAGGCCG |  |
| GBLPF | CAAGTACACCATTGGCGAGC | qRT-PCR primers of reference gene |
| GBLPR | CTTGCAGTTGGTCAGGTTCC |  |
| QpFAX1F | GTGCTAAGCCTCCAGGAGTGGAA | qRT-PCR primers of FAX1 expression in transgenetic algae |
| QpFAX1R | ACGTTGTAGGCGCAGAAGAGC |  |
| QpFAX2F | GTGCCTACATCTACTTCACC | qRT-PCR primers of FAX2 expression in transgenetic algae |
| QpFAX2R | TCTTCTCGAACCGCCTGTACATC |  |
| ScFAX1F BamHI | GGATCCATGGCCGGCCCCGCCCGGCAC | Primers used for construction of plasmid pYES2-CrFAX1 |
| ScFAX1R XbaI | TCTAGACTACTCGGCCTTGCCG |  |
| ScFAX2F BamHI | GGATCCATGTATGACTTTTGCTTTTCGCCC | Primers used for construction of plasmid pYES2-CrFAX2 |
| ScFAX2R EcoRI | GAATTCCTAGTGCGCCTTGCCGT |  |
| QLACS1F | CGTCGGATACTGGCAGGGTG | qRT-PCR primers of LACS1 expression in transgenetic algae |
| QLACS1R | ATAAGCCGGGACAGGAAGC |  |
| QLACS2F | CCAAGTTTCCGCACATTCCG | qRT-PCR primers of LACS2 expression in transgenetic algae |
| QLACS2R | CAGCCACTCCTTGCAGTTTGGACG |  |
