# Supplementary material for: Characterization of Fatty Acid Exporters involved in fatty acid transport for oil accumulation in the green alga Chlamydomonas reinhardtii
Source: Biotechnol Biofuels. 2019 Jan 12;12:14. doi: 10.1186/s13068-018-1332-4 (PMC6330502; doi:10.1186/s13068-018-1332-4)
Supplement: Supplementary file 2 — Additional file 2: Table S2. All gene names and accession numbers presented in this study. [file 13068_2018_1332_MOESM2_ESM.docx]

**Additional file 2: Table S2**

| **species** | **gene Name** | **accession No.** |
| --- | --- | --- |
| *Chlamydomonas reinhardtii* | CrFAX1 | XP_001702596.1 |
| *Chlamydomonas reinhardtii* | CrFAX2 | XP_001696048.1 |
| *Volvox carteri* | VcFAX | XP_002948244.1 |
| *Gonium pectorale* | GPFAX1 | KXZ53836.1 |
| *Gonium pectorale* | GPFAX2 | KXZ50213.1 |
| *Ectocarpus siliculosus* | EsFAX | CBJ26023.1 |
| *Micromonas pusilla* | MpFAX | XP_003062132.1 |
| *Chlorella variabilis* | CvFAX | XP_005843190.1 |
| *Arabidopsis thaliana* | AtFAX1 | NP_567046.1 |
| *Arabidopsis thaliana* | AtFAX2 | NP_565892.1 |
| *Arabidopsis thaliana* | AtFAX3 | [NP_566866.1](https://www.ncbi.nlm.nih.gov/protein/18407583?report=genbank&log$=prottop&blast_rank=1&RID=7SV73VJW015) |
| *Arabidopsis thaliana* | AtFAX4 | [NP_564422.1](https://www.ncbi.nlm.nih.gov/protein/18398785?report=genbank&log$=prottop&blast_rank=1&RID=7SVBZ836015) |
| *Arabidopsis thaliana* | AtFAX5 | NP_564579.1 |
| *Arabidopsis thaliana* | AtFAX6 | [NP_188687.1](https://www.ncbi.nlm.nih.gov/protein/18407583?report=genbank&log$=prottop&blast_rank=1&RID=7SV73VJW015) |
| *Arabidopsis thaliana* | AtFAX7 | [NP_180192.1](https://www.ncbi.nlm.nih.gov/protein/18398785?report=genbank&log$=prottop&blast_rank=1&RID=7SVBZ836015) |
| *Brassica napus* | BnaFAX1 | [XP_013652026.1](https://www.ncbi.nlm.nih.gov/protein/923675617?report=genbank&log$=prottop&blast_rank=1&RID=7STYJJEV015) |
| *Brassica napus* | BnaFAX2 | [XP_013707314.1](https://www.ncbi.nlm.nih.gov/protein/923675617?report=genbank&log$=prottop&blast_rank=1&RID=7STYJJEV015) |
| *Brassica napus* | BnaFAX3 | [XP_013661405.1](https://www.ncbi.nlm.nih.gov/protein/923675617?report=genbank&log$=prottop&blast_rank=1&RID=7STYJJEV015) |
| *Brassica napus* | BnaFAX4 | [XP_013708154.1](https://www.ncbi.nlm.nih.gov/protein/923675617?report=genbank&log$=prottop&blast_rank=1&RID=7STYJJEV015) |
| *Brassica napus* | BnaFAX5 | CDY25185.1 |
| *Brassica napus* | BnaFAX6 | [XP_013673315.1](https://www.ncbi.nlm.nih.gov/protein/923675617?report=genbank&log$=prottop&blast_rank=1&RID=7STYJJEV015) |
| *Brassica napus* | BnaFAX7 | CDX76725.1 |
| *Glycine max* | GmFAX1 | [XP_003554154.1](https://www.ncbi.nlm.nih.gov/protein/356571992?report=genbank&log$=prottop&blast_rank=1&RID=7T1V5MTU015) |
| *Glycine max* | GmFAX2 | [NP_001240196.1](https://www.ncbi.nlm.nih.gov/protein/358248790?report=genbank&log$=prottop&blast_rank=1&RID=7T1ZN98E015) |
| *Glycine max* | GmFAX3 | [NP_001235243.1](https://www.ncbi.nlm.nih.gov/protein/351723771?report=genbank&log$=prottop&blast_rank=2&RID=7T2447WZ015) |
| *Glycine max* | GmFAX4 | [XP_003552314.1](https://www.ncbi.nlm.nih.gov/protein/356568226?report=genbank&log$=prottop&blast_rank=1&RID=7T2B9N1U014) |
| *Glycine max* | GmFAX5 | XP_003534642.1 |
| *Glycine max* | GmFAX6 | XP_003529052.1 |
| *Solanum lycopersicum* | SlFAX1 | NP_001307153.1 |
| *Solanum lycopersicum* | SlFAX2 | XP_004236577.1 |
| *Solanum lycopersicum* | SlFAX3 | XP_004244130.1 |
| *Solanum lycopersicum* | SlFAX4 | XP_004248379.1 |
| *Solanum lycopersicum* | SlFAX5 | XP_004246287.1 |
| *Solanum lycopersicum* | SlFAX6 | XP_004251316.1 |
| *Oryza sativa* | OsFAX1 | XP_015635421.1 |
| *Oryza sativa* | OsFAX2 | [XP_015641040.1](https://www.ncbi.nlm.nih.gov/protein/1002273457?report=genbank&log$=prottop&blast_rank=1&RID=7T4MUTTZ014) |
| *Oryza sativa* | OsFAX3 | XP_015621232.1 |
| *Oryza sativa* | OsFAX4 | EAZ27528.1 |
| *Oryza sativa* | OsFAX5 | XP_015628725.1 |
