# Supplementary material for: Characterization of Fatty Acid Exporters involved in fatty acid transport for oil accumulation in the green alga Chlamydomonas reinhardtii
Source: Biotechnol Biofuels. 2019 Jan 12;12:14. doi: 10.1186/s13068-018-1332-4 (PMC6330502; doi:10.1186/s13068-018-1332-4)

**Additional file 3: Figure S1**

The protein sequences Alignment of CrFAX1, CrFAX2 and AtFAX1. The four transmembrane domains of each protein were marked with red, yellow or green square frames.


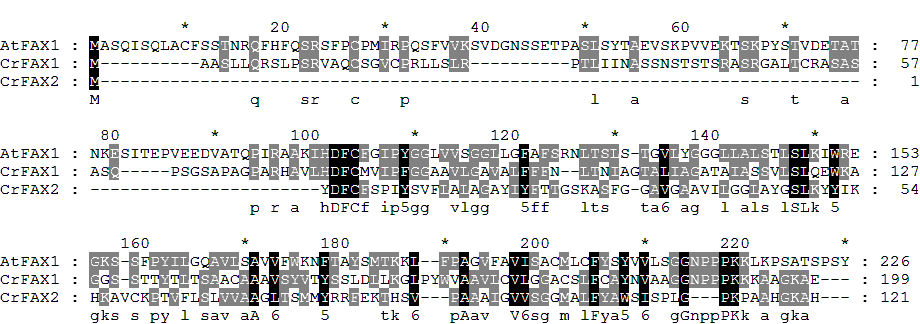

Supplement: Supplementary file 3 — Additional file 3: Figure S1. The protein sequences Alignment of CrFAX1, CrFAX2 and AtFAX1. [file 13068_2018_1332_MOESM3_ESM.docx]
