# Supplementary material for: Characterization of Fatty Acid Exporters involved in fatty acid transport for oil accumulation in the green alga Chlamydomonas reinhardtii
Source: Biotechnol Biofuels. 2019 Jan 12;12:14. doi: 10.1186/s13068-018-1332-4 (PMC6330502; doi:10.1186/s13068-018-1332-4)

Additional file 4: Figure S2

The conserved motifs prediction was constructed using MEME. Both CrFAXs contain motif1, which may be functional motif.


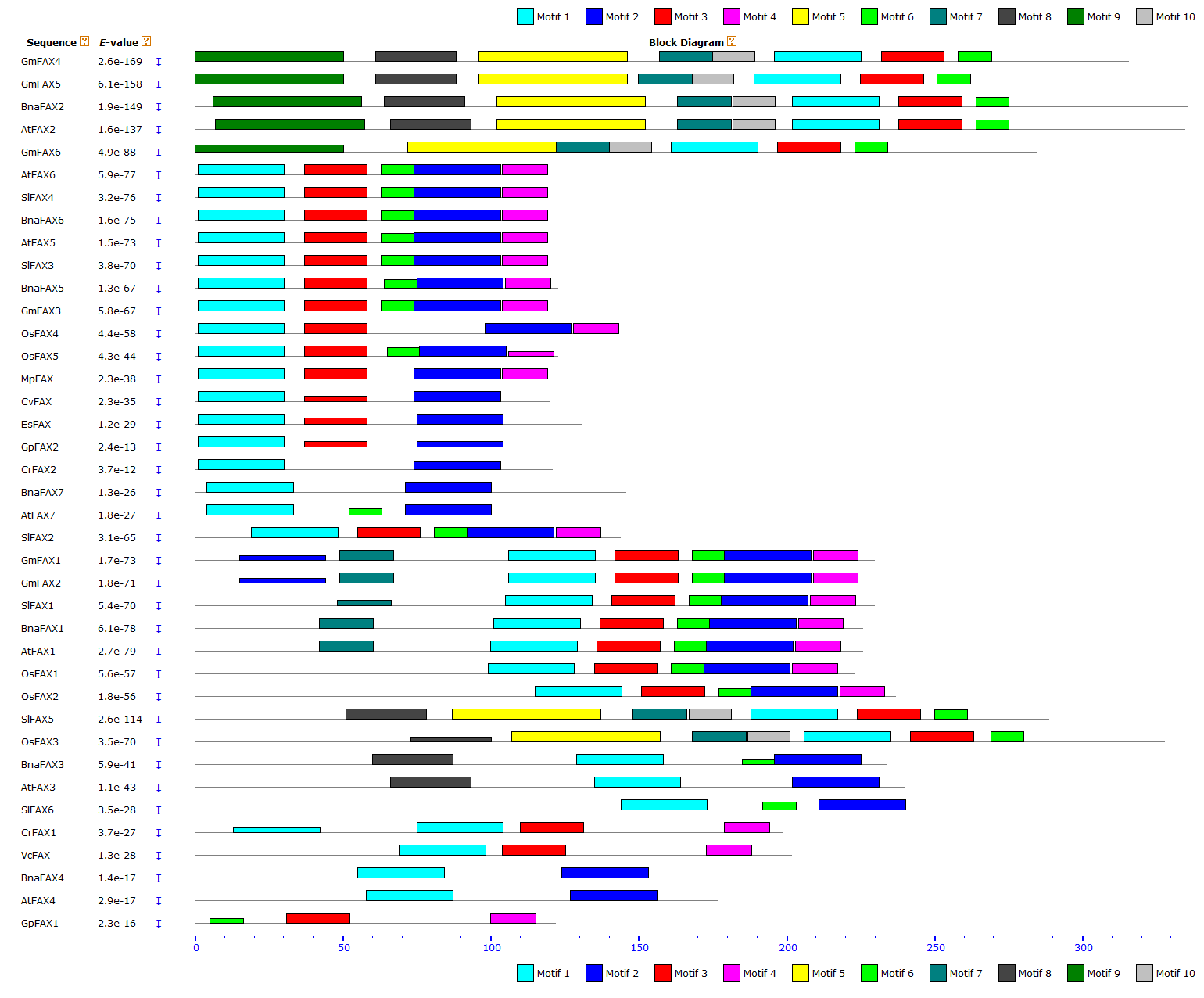

Supplement: Supplementary file 4 — Additional file 4: Figure S2. The conserved motifs prediction was constructed using MEME. [file 13068_2018_1332_MOESM4_ESM.docx]
