# Supplementary material for: Characterization of Fatty Acid Exporters involved in fatty acid transport for oil accumulation in the green alga Chlamydomonas reinhardtii
Source: Biotechnol Biofuels. 2019 Jan 12;12:14. doi: 10.1186/s13068-018-1332-4 (PMC6330502; doi:10.1186/s13068-018-1332-4)
Supplement: Supplementary file 5 — Additional file 5: Table S3. The information of conserved motifs. [file 13068_2018_1332_MOESM5_ESM.docx]

Additional file 5: Table S3 The information of conserved motifs

| **MOTIF** | **WIDTH** | **BEST POSSIBLE MATCH** |
| --- | --- | --- |
| 1 | 29 | HDFCFTIPYGMLLMVGGFMGYMKKGSTAS |
| 2 | 29 | VMGQRYMQTRKIMPWGIVAIISACMTCFY |
| 3 | 21 | GGALLALGYLSLKAWRKGKNS |
| 4 | 15 | YNIATGGNPPPKKKE |
| 5 | 50 | QEAWKQTLDTFKEQAEKMQGVSQEAYEVYSQKAMEILKDTSEQLKIQADK |
| 6 | 11 | GQTVCAAILFW |
| 7 | 18 | IGDETKEYLSYAADNSPE |
| 8 | 27 | RNRLTVCFTANHEDSEHKDVEVEKERD |
| 9 | 50 | MMSFRNDNVTVLNPKHSHPHASLPFNRFPHLPNFHHHLKPRAVNACVPPN |
| 10 | 14 | VKEIVETFTSPPDD |
