# Supplementary material for: Characterization of Fatty Acid Exporters involved in fatty acid transport for oil accumulation in the green alga Chlamydomonas reinhardtii
Source: Biotechnol Biofuels. 2019 Jan 12;12:14. doi: 10.1186/s13068-018-1332-4 (PMC6330502; doi:10.1186/s13068-018-1332-4)

**Additional file 6: Figure S3. 3D modeling prediction of CrFAXs and AtFAXs**

The 3D modellings of CrFAX1, CrFAX2, AtFAX1, AtFAX2 and AtFAX3 were structured using Phyre server.


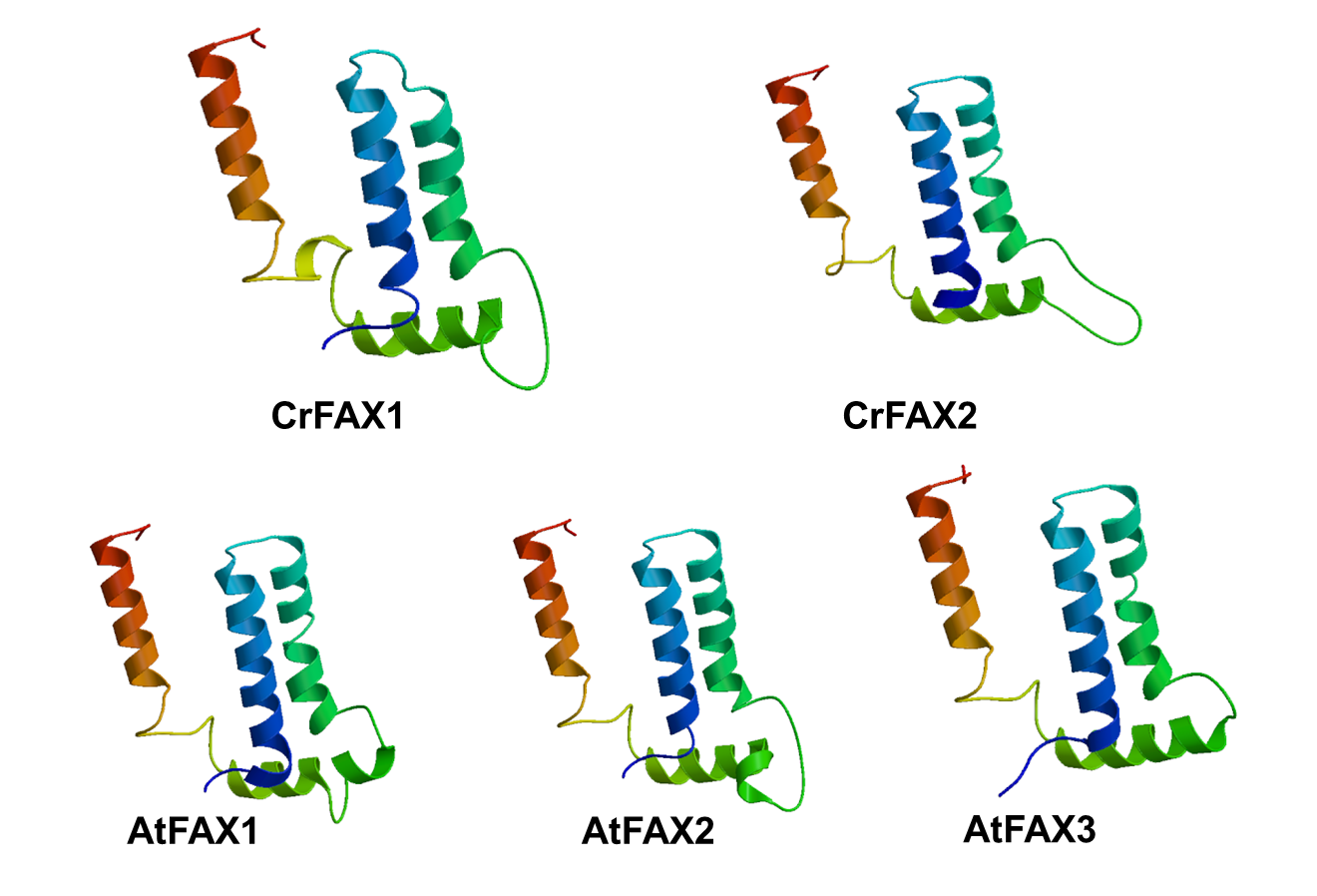

Supplement: Supplementary file 6 — Additional file 6: Figure S3. 3D modeling prediction of CrFAXs and AtFAXs. [file 13068_2018_1332_MOESM6_ESM.docx]
