# Supplementary material for: Characterization of Fatty Acid Exporters involved in fatty acid transport for oil accumulation in the green alga Chlamydomonas reinhardtii
Source: Biotechnol Biofuels. 2019 Jan 12;12:14. doi: 10.1186/s13068-018-1332-4 (PMC6330502; doi:10.1186/s13068-018-1332-4)

**Additional file 7: Figure S4. Screening mutants by using PCR amplification.**

Screening the single colony of transformed *C. reinhardtii* cell from plates with paromomycin resistance and extracting genomic DNA. Two pairs of primer were designed to amplify APHVIII gene and PPsaD promoter. The primers APFAXsF and APFAXsR were used to amplify the 3' end of APHVIII gene(360 bp). Another pair of primer PPFAXsF and PPFAXsF was used to amplify the 5' end of the GFP promoter PPsaD (380 bp) (Neupert,et.2009)


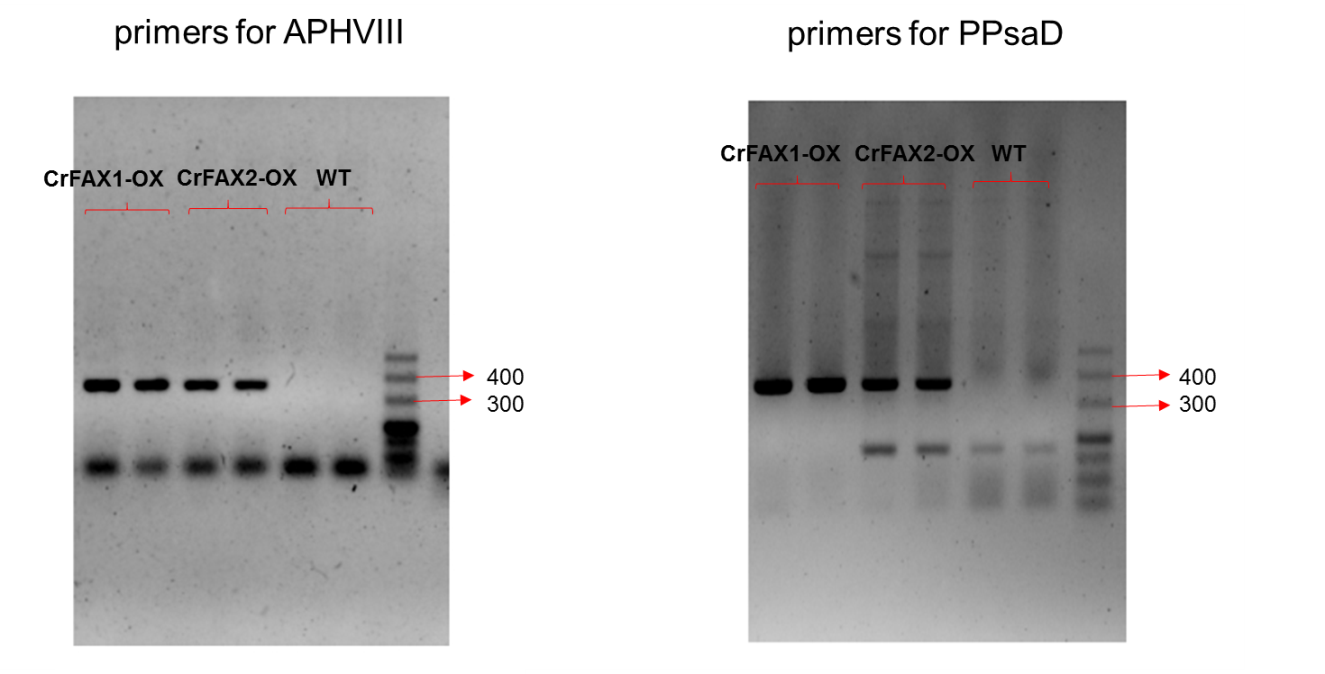

Supplement: Supplementary file 7 — Additional file 7: Figure S4. Screening mutants by using PCR amplification. [file 13068_2018_1332_MOESM7_ESM.docx]
