# Supplementary material for: Characterization of Fatty Acid Exporters involved in fatty acid transport for oil accumulation in the green alga Chlamydomonas reinhardtii
Source: Biotechnol Biofuels. 2019 Jan 12;12:14. doi: 10.1186/s13068-018-1332-4 (PMC6330502; doi:10.1186/s13068-018-1332-4)
Supplement: Supplementary file 8 — Additional file 8: Table S4. QC items for each sample. [file 13068_2018_1332_MOESM8_ESM.docx]

**Additional file 8: Table S4 QC items for each sample**

| Sample | Clean Read1 Q20(%)≥90 | Clean Reads≥ 20 (M) | Gene Unique Mapping Ratio(%)≥ 80 | Genome Mapping Ratio(%)≥ 50 |
| --- | --- | --- | --- | --- |
| CrFAX1ox1 | 98.0 (Y) | 23.03 (Y) | 90.28 (Y) | 96.03 (Y) |
| CrFAX1ox2 | 96.3 (Y) | 22.61 (Y) | 91.15 (Y) | 95.56 (Y) |
| CrFAX2ox1 | 97.9 (Y) | 22.64 (Y) | 91.19 (Y) | 95.65 (Y) |
| CrFAX2ox2 | 97.8 (Y) | 23.01 (Y) | 90.89 (Y) | 96.13 (Y) |
| WT1 | 96.8 (Y) | 22.70 (Y) | 90.32 (Y) | 77.12 (Y) |
| WT2 | 97.9 (Y) | 23.27 (Y) | 90.25 (Y) | 77.1 (Y) |
