# Supplementary material for: Characterization of Fatty Acid Exporters involved in fatty acid transport for oil accumulation in the green alga Chlamydomonas reinhardtii
Source: Biotechnol Biofuels. 2019 Jan 12;12:14. doi: 10.1186/s13068-018-1332-4 (PMC6330502; doi:10.1186/s13068-018-1332-4)
Supplement: Supplementary file 9 — Additional file 9: Table S5. The lipid metabolism related genes regulated in CrFAXs overexpression strains. [file 13068_2018_1332_MOESM9_ESM.docx]

**Additional file 9: Table S5 The lipid metabolism related genes regulated in CrFAXs overexpression strains**

| **Gene ID** | **Pathways ID** | **Annotation** | **Log_2_OXs/WT** |
| --- | --- | --- | --- |
| XP_001702947.1 | K01897 | Long Chain Acyl-CoA Synthetase 1 | 0.79 |
| XP_001690836.1 | K01897 | Long Chain Acyl-CoA Synthetase 2 | 1.13 |
| XP_001694618.1 | K00507 | stearoyl-CoA desaturase-1 | 0.88 |
| XP_001696619.1 | K10782 | acyl-ACP thioesterase | 1.10 |
| XP_001689723.1 | K10525 | allene oxide cyclase, AOC | 1.31 |
| XP_001701720.1 | K00472 | prolyl hydroxylase, PH | -1.61 |
