# Supplementary material for: Characterization of Fatty Acid Exporters involved in fatty acid transport for oil accumulation in the green alga Chlamydomonas reinhardtii
Source: Biotechnol Biofuels. 2019 Jan 12;12:14. doi: 10.1186/s13068-018-1332-4 (PMC6330502; doi:10.1186/s13068-018-1332-4)

**Additional file 10: Figure S5. Distribution of most abundant Gene ontology (GO) terms assigned to the** **CrFAX1-OX vs WT (a) and CrFAX2-OX vs WT (b).**

Within the “biological process” category of the GO analysis, there were 301 and 256 regulated genes, respectively. In the “cellular component” category, there were 422 and 350 regulated genes. In the “molecular function” category, 218 and 172 genes were regulated in CrFAX1-OX and CrFAX2-OX comparing with WT.


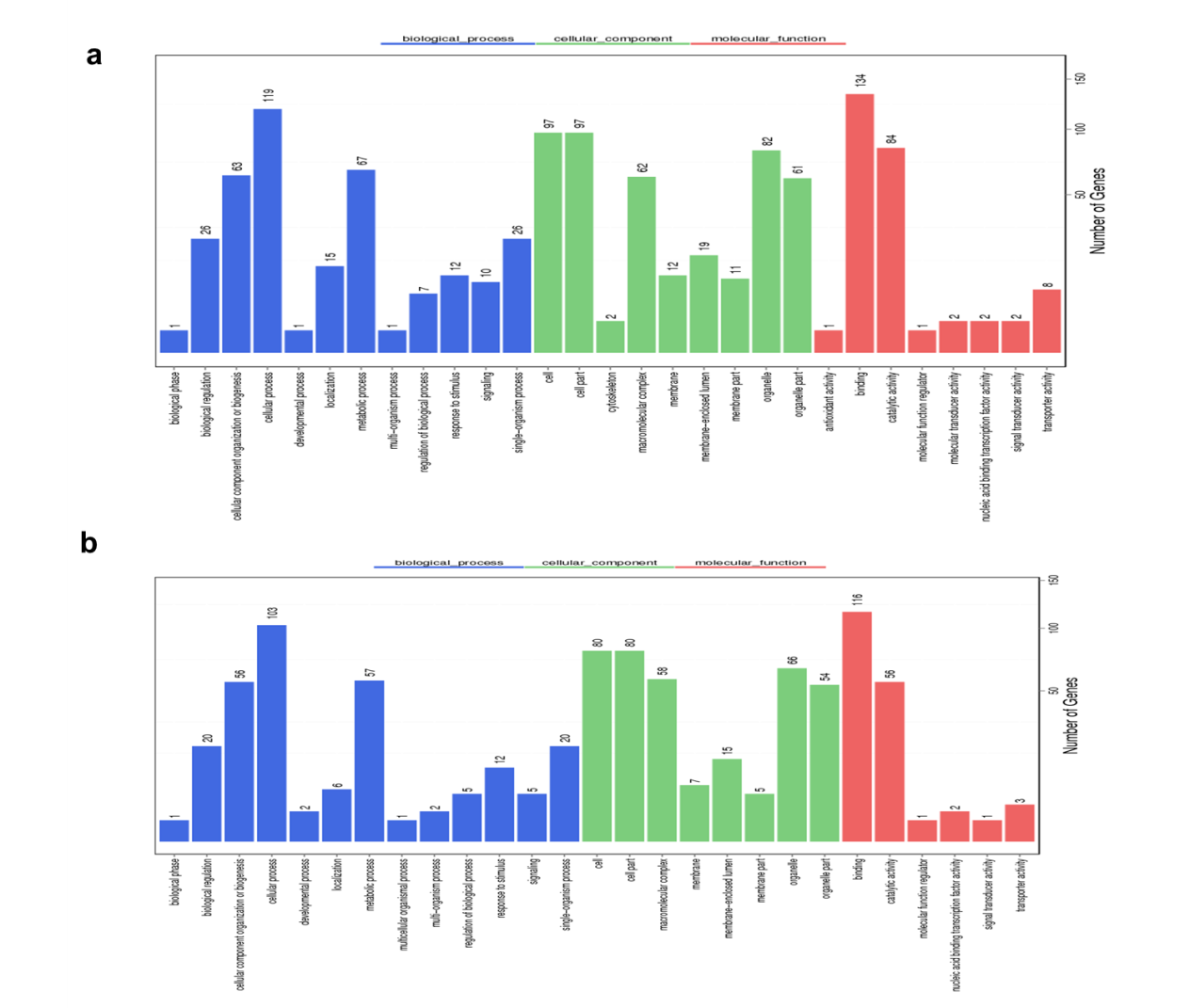

Supplement: Supplementary file 10 — Additional file 10: Figure S5. Distribution of most abundant Gene ontology (GO) terms assigned to the CrFAX1 vs WT (a) and CrFAX2 vs WT (b). [file 13068_2018_1332_MOESM10_ESM.docx]
