# Supplementary material for: Characterization of Fatty Acid Exporters involved in fatty acid transport for oil accumulation in the green alga Chlamydomonas reinhardtii
Source: Biotechnol Biofuels. 2019 Jan 12;12:14. doi: 10.1186/s13068-018-1332-4 (PMC6330502; doi:10.1186/s13068-018-1332-4)

**Additional file 11: Figure S6. KEGG classification on DEGs for CrFAX1-OX vs WT (a) and CrFAX2-OX vs WT (b).** KEGG metabolic pathway analysis revealed that 71 DEGs were distributed among 15 and 17 metabolic pathways for the regulated genes, respectively**.**


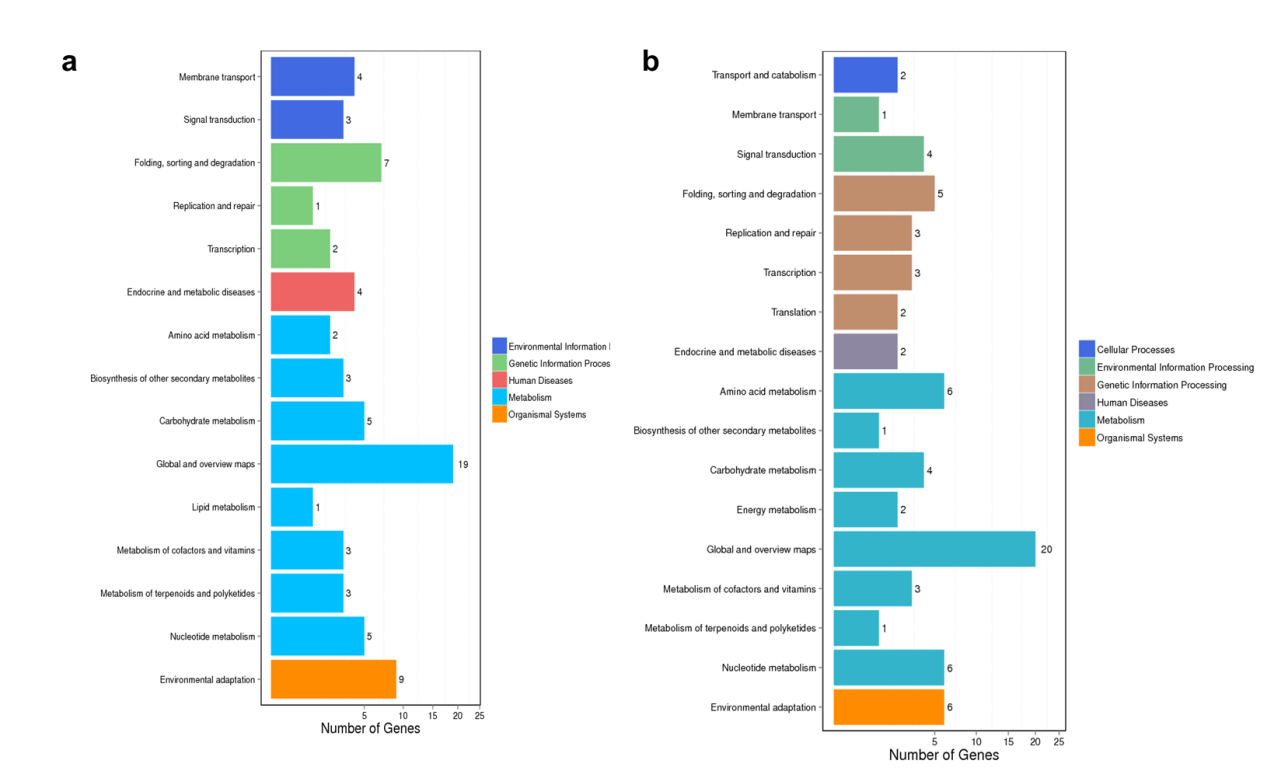

Supplement: Supplementary file 11 — Additional file 11: Figure S6. KEGG classification on DEGs for CrFAX1 vs WT (a) and CrFAX2 vs WT (b). [file 13068_2018_1332_MOESM11_ESM.docx]
