# Supplementary material for: Characterization of Fatty Acid Exporters involved in fatty acid transport for oil accumulation in the green alga Chlamydomonas reinhardtii
Source: Biotechnol Biofuels. 2019 Jan 12;12:14. doi: 10.1186/s13068-018-1332-4 (PMC6330502; doi:10.1186/s13068-018-1332-4)
Supplement: Supplementary file 12 — Additional file 12: Figure S7. Substrate utilization profiles of yeast strains expressing crfax1 and crfax2. [file 13068_2018_1332_MOESM12_ESM.docx]

**
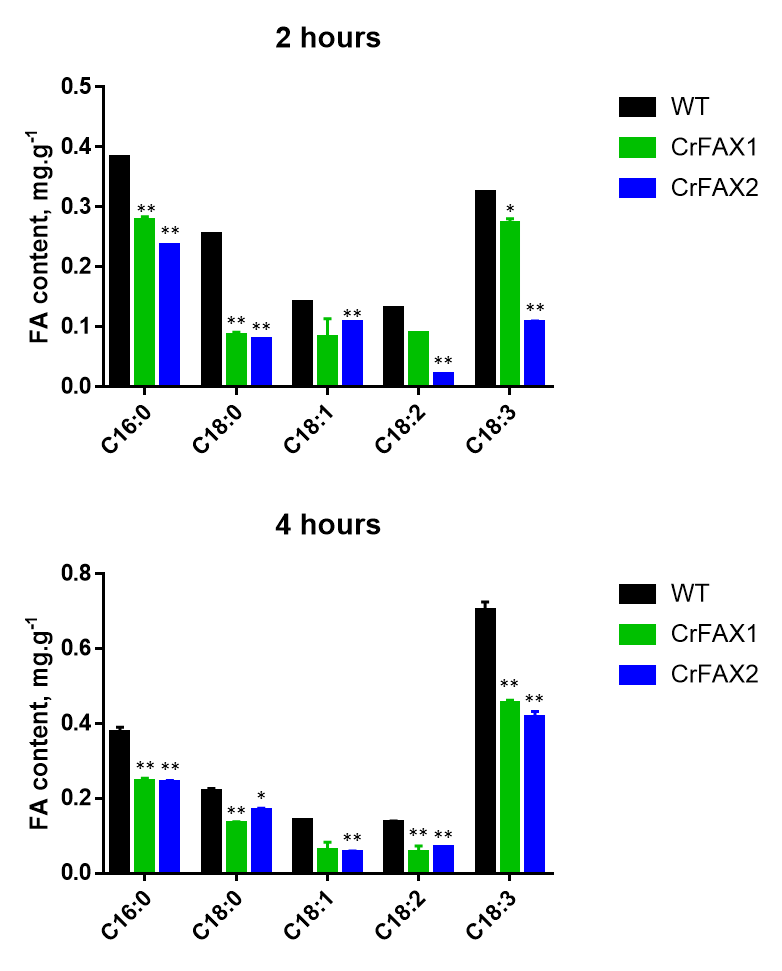
**

**Additional file 12: Figure S7 Substrate utilization profiles of yeast strains expressing *crfax1* and *crfax2*.** Cells expressing *crfax1* and *crfax2* were named pYES2-CrFAX1 and pYES2-CrFAX2, respectively. BY4741 transformed with a blank pYES2 vector was used as the control strain. The growth of yeast cells in liquid medium with different FAs as the carbon sources was observed and detected after growing 2 h (**a**) and 4 h (**b**), respectively. The results are shown as the mean expression ± standard deviation (SD) of three independent experiments. Student’s *t*-test, ^*^*P*<0.05, ^**^*P*<0.01.

**Methods**

Cells expressing *crfax1* and *crfax2* were named pYES2-CrFAX1 and pYES2-CrFAX2, respectively. The transformants and BY4741 with blank pYES2 were grown to mid-log phase in dropout medium containing 1% dextrose, then harvested, and washed with 2 M sorbitol for two times. Cells were re-suspended in fresh dropout medium containing 2% galactose and cultured for 4 h to induce *crfax* expression. And then 100 μmol/L C16:0, C18:0, C18:1, C18:2 and C18:3, 1% tergitol were added to the culture and then the cultures were incubated for 2 h and 4 h, respectively. The cultures were harvested and the fatty acids were measured with gas chromatography.
